# Assessing the Role of AI-Based Smart Sensors in Smart Cities Using AHP and MOORA

**DOI:** 10.3390/s23010494

**Published:** 2023-01-02

**Authors:** Habib Ullah Khan, Shah Nazir

**Affiliations:** 1 Department of Accounting and Information Systems, College of Business and Economics, Qatar University, Doha 2713, Qatar; 2 Department of Computer Science, University of Swabi, Swabi 23430, Pakistan

**Keywords:** the Internet of Everything, smart sensors, artificial intelligence

## Abstract

We know that in today’s advanced world, artificial intelligence (AI) and machine learning (ML)-grounded methodologies are playing a very optimistic role in performing difficult and time-consuming activities very conveniently and quickly. However, for the training and testing of these procedures, the main factor is the availability of a huge amount of data, called big data. With the emerging techniques of the Internet of Everything (IoE) and the Internet of Things (IoT), it is very feasible to collect a large volume of data with the help of smart and intelligent sensors. Based on these smart sensing devices, very innovative and intelligent hardware components can be made for prediction and recognition purposes. A detailed discussion was carried out on the development and employment of various detectors for providing people with effective services, especially in the case of smart cities. With these devices, a very healthy and intelligent environment can be created for people to live in safely and happily. With the use of modern technologies in integration with smart sensors, it is possible to use energy resources very productively. Smart vehicles can be developed to sense any emergency, to avoid injuries and fatal accidents. These sensors can be very helpful in management and monitoring activities for the enhancement of productivity. Several significant aspects are obtained from the available literature, and significant articles are selected from the literature to properly examine the uses of sensor technology for the development of smart infrastructure. The analytical hierarchy process (AHP) is used to give these attributes weights. Finally, the weights are used with the multi-objective optimization on the basis of ratio analysis (MOORA) technique to provide the different options in their order of importance.

## 1. Introduction

It has been demonstrated that the employment of AI- and ML-based algorithms in the creation of automated systems is quite effective. In smart buildings, tracking devices and sensors generate a large amount of data. One of the cutting-edge components that can enhance intelligent urban infrastructure is big-data analytics. Intelligent IoT devices, automated systems, and sensing equipment continuously gather massive amounts of data in smart cities. The application of data analytics and machine learning techniques determines how accurate a forecast is.

An analysis of the existing approaches for detection in smart buildings was presented through investigation. Contents cover monitoring applications in intelligent cities, sensing platforms, and technical issues related to these technologies. A variety of applications and the technical difficulties involved with these applications are covered in an attempt to provide a comprehensive understanding of how sensing technologies function in smart cities. The information provided in this study attempts to connect these gaps, because some of these methodologies fall under the purview of distinct subject areas. This optimistic overview can assist professionals in recognizing how sophisticated detection can contribute to the development of smart cities [1]. Ramírez-Moreno et al. [2] examined the various sensors that are frequently employed in projects to build smart cities. There are insights regarding various applications and communication technologies, as well as the primary potential and difficulties encountered when converting to a smart city. In the end, this study is about more than just smart urban infrastructure; it is also about how these new digitalization and monitoring advancements enhance living conditions. Smarter societies are those that invest in, socialize with, and adapt to these innovations in conformity with local and regional societal requirements and ideals.

Smart cities are playing a very important role in providing people with quick services. For handling the hurdles of the heterogeneity of the sensors, Fazio et al. [3] developed a novel paradigm that is capable of dual abstraction of sophisticated sensing networks and the knowledge they gather. Offering a global solution that is adaptable and scalable is a crucial component of the proposed approach. The architecture framework utilizes the Contiki operating system and is focused on Sensor Web Enablement standard protocols to deploy the Internet of Things. Channi and Kumar [4] drew attention to the necessity of sensing devices in smart cities for remotely controlled systems. The sophisticated temperature sensors were described in depth. With illustrations such as water management systems, sustainable energy, lighting control mechanisms, and sewage treatment, the implementations of super-clever temperature sensors in smart buildings were also covered. Cities with smart buildings can supply vital services more swiftly and effectively due to an array of sensors, camera systems, cabling, wireless connections, and data centers.

Due to various unique and interesting technologies, such as the Internet of Things, smart sensing-based systems can be employed for peoples’ well-being and progress. The efficient usage of such methodologies can provide people with basic needs at their doorstep. The main goals of this suggested approach are:

- to analyze the impacts of sensor-grounded systems on the lives of human beings;
- to consider the role of modern technology in the implementation of intelligent IoT-based systems;
- to extract useful parameters and select significant parameters from the available techniques;
- to use AHP to calculate the weights of these attributes and to utilize the MOORA process to carry out the ranking of options.

This article is organized as follows: Section 1 provides an overview of the study. A summary of the currently used research methodologies is provided in Section 2. The study’s executed technique is provided in Section 3. The study’s findings are the focus of Section 4. A general summary of the entire research is presented in Section 5.

## 2. Related Work

The main goal of the proposed arrangement is to provide a more intelligent approach to waste management, using an intuitive sensor. The sensor assists in determining the amount of rubbish in the trashcan and real-time data gathered from numerous trashcans spread out in different locations. Smart cities can be considered when implementing this technology. To locate the quickest and cheapest route to pick up the trash, the drivers would utilize a smartphone application. The statistics would be refreshed on the website whenever the trash is gathered [5]. Data science and machine learning insights are equally important to computing and cannot be disregarded. Quasim et al. [6] performed a study on the basics of smart cities and sectors in smart cities Data analytics methods are presented in the present study. The tracking sensors and gadgets in smart buildings produce enormous amounts of data. One of the advanced components that can enhance intelligent urban amenities is big data analytics. Large amounts of data are continuously collected in smart cities from sensing devices, automated equipment, and sophisticated IoT devices. The methods of data analytics and machine learning techniques determine how precise a forecast will be. Sumi and Ranga [7] updated the newest reports in this field of study and identified some significant problems with the sensor-enabled Internet-of-Things-grounded systems for smart cities. In recent years, the Internet of Things has also drawn interest that was previously unheard of. It links thousands of sensory gadgets to the Internet, which can be utilized to economically and successfully manage urban areas. The characteristics of a sensor-enabled Internet of Things were noted in the suggested research, highlighting their possibilities in the creation of a smart city.

Ji [8] suggested an approach for multi-sensor fault detection built on smart detection architecture. To compute the Kalman filter approximation result with state constraints, the technique first builds formulas that take into account physical limitations. This is done for data validation and fault diagnostics. The highly decentralized nature of the suggested method enables independent computations by several monitoring stations. An in-depth discussion of the methodology development procedure is included in the document, along with information on how the program’s modeling, testing, and maintenance were used to confirm the application’s parallelism, correctness, and durability. Most people think of the Internet of Things as a network of connected ”things” in a smart urban environment. It can also be seen as a gathering of information that is exchanged among several systems and cutting-edge sensors, then examined and applied to various parts of the modern city. This study examines the similarities between a layered network design and a “layered digitalization structure” that is utilized in smart buildings to gather and exchange massive data with the help of a cloud platform. The discussion of the Internet of Things is carried out from the perspective of particular devices and services, such as automation and smart monitoring paradigms [9]. 

The notion of intelligent cities is becoming a reality thanks to the Internet-of-Things-grounded smart and intelligent sensors. Vakula and Kolli [10] introduced a parking solution for smart cities that utilizes the Internet of Things. The suggested parking paradigm has an IoT subsystem installed on site to manage the free parking spots. A browser interface is made available for public parking reservations. IoT is solving the most prevalent urban issues, such as available parking spaces and traffic congestion.

A blockchain is preserved, in which the entries of all valid connected systems are stored to safeguard against future alterations of data acquired by digital sensors. That article’s goal was to identify and address the many hazards brought on by an intrusion at different stages. To that end, it implemented a safe and secure e-voting technique using IoT devices that utilized blockchain technology. Additionally, the suggested system was examined on the basis of several security criteria, including message tampering, DoS and DDoS attacks, and authorization latency, to verify it [11]. Shamsir et al. [12] provided a summary of the most recent embedded sensors that can be extremely important in the planning of a smart city. Asset usage and climate change have been greatly impacted by industrialization and fast urbanization. That article’s goal was to present a succinct overview of detecting technology in various niche applications, including defense, farming, food hygiene, and environmental control. 

The present work offers a new direction for research by examining the advent of low-power sensor devices and related difficulties. The designed scheme includes several in-vehicle sensing devices that track a variety of variables, including speed, distance, and protective measures such as seatbelts, smart locks, airbags, and handbrakes. The car can adjust to diverse scenarios using the historical data gathered and the real-time data saved in the cloud. Additionally, users of Google Assistant may lock and unlock, start and stop, set up an alarm, and carry out many programmed activities, such as checking for low fuel or insurance coverage. The suggested real-time IoT-enabled vehicle system is capable of detecting collisions and modifying itself in response to changing circumstances [13].

Smart and portable sensors are revolutionizing the field of smart cities. Morello et al. [14] emphasized the development of technological innovations and their uses in smart urban areas and power grids. Electric grid and municipal services are provided with a significant boost due to the employment of sophisticated sensor systems and intelligent transducers. The ultimate goal of this article is to present readers with a better grasp of the existing approaches in this area by discussing diverse uses and outlining additional features and concepts for sensors and detecting platforms used in green infrastructure, power networks, and electric grids. De Paz et al. [15] proposed an adaptable design that centralized the administration of clever management and general lighting to conserve lights and preserve the best possible visual performance in lit spaces. The design combined artificial intelligence (AI) and statistical approaches to conduct this monitoring, including artificial neural networks (ANN) and multi-agent systems (MAS). Utilizing a modular approach, it would optimize energy usage and costs, while being completely compatible with conventional street lighting. The framework is still being developed after effectively passing tests and receiving validation. 

The inability to locate unoccupied parking spaces is an issue that frequently arises in our cities. People who commute to the workplace are seeking a spot to park, which clogs the roadway. A navigation-and-reservation-grounded parking suggestion system was devised for smart cities in the research that is being considered in this article. The suggested approach entails the creation of tiny gadgets that transmit data to the internet using Internet of Things (IoT) innovation. A technique is used to locate the available parking place that is nearest to the present location. A variety of cases are evaluated using the suggested approach, and precise results are produced [16]. Various applications of the IoT exist in different domains [17,18].

Kang et al. [19] classified home automation operations, examined the various kinds of sensors utilized, and then employed the sensor tree to calculate the relevance of each sensor. The motion detector has a strong relevance score as a consequence, and seven intelligent home applications utilize it. Numerous detectors and intelligent items are utilized during the fundamental shift from the connected home to the modern city to provide services to users. Recognizing and analyzing the redundant sensors used by present smart home services is vital for designing the future digital city. Although smart cities strive for further effective asset management with smart metering, smart transportation, etc., another strategy may be used. Decent healthcare solutions for residents may be delivered, due to the extensive sensor employment in smart cities. In the proposed method, it was demonstrated how this may be accomplished by letting patients communicate with metropolitan devices. Patients with breathing issues use their cell phones to discover the shortest and healthiest path to their location [20]. Csáji et al. [21] studied a Budapest smart city project proposal that makes use of a cloud-grounded analytical subsystem and different sensors located on the streetlight infrastructure. The device combines and mathematically analyzes the data while the deployed wireless multi-sensor connection collects knowledge about particular stressors. The unit can extend observations in space and time, and manage conflicting, absent, and noisy data. The resultant repository, which employs geometrical visualizations, can act as a hub for public infrastructure knowledge.

Based on the employment of intelligent sensors in smart cities, efficient facilities can be provided to citizens. The authors of [22] suggested an intelligent manhole cover management system (IMCS) for smart urban areas, utilizing edge computing. Every manhole cover has a distinct frequency identification label with orientation and vibrating detectors, and a Narrowband Internet of Things is utilized for interaction. Based on the data gathered, edge computing platforms communicate with the associated managers via handheld devices. The suggested IMCS’s great productivity was demonstrated by practical implementation in Hangzhou, China’s Xiasha District. It successfully decreased the typical maintenance costs, potentially increasing both manhole cover safety and public safety. Duangsuwan et al. [23] developed sophisticated air pollution sensors to track the state of the atmosphere in smart cities. The study suggested smart detectors that track ozone (O3), carbon monoxide (CO), carbon dioxide (CO_2_), noise level (dB), particulate matter (PM 10), or dust, respectively. This sensor technology is a low-power wide-area network approach (LPWAN). The investigation looked at the assessment in Bangkok, and the findings showed the air quality index (AQI) using the Narrowband Internet of Things (NB-IoT). The applications of smart sensors are available in different areas [24,25,26].

## 3. Methodology

The use of smart sensors plays a very crucial role in the prediction and effective decision-making architectures. They are very helpful in the development of intelligent embedded systems for achieving high accuracy in complex and difficult tasks, such as power management, health-related decision making, and educational activities. Their usage is gaining increasing attention with the implementation of information and communication technologies.

With the rise of smart cities, the research seeks to investigate the security issues that are introduced by suspicious network assaults in human resources administration. Initially, the privacy of the data physics platform is successfully assessed using the Stackelberg game theory methodological approach to characterize the relationship between sensing devices and smart jammers. To secure the confidentiality of embedded systems, a denoise autoencoder machine architecture, which may be employed in human resource management using demographic information, is provided. In the end, its performance is examined and modeled [27]. Doran et al. [28] outlined how individuals might act as human sensors to provide additional, alternative, and alternative data resources for smart cities. That article offers a way of extracting from social media posts the opinions that could be pertinent to projects involving smart cities, using a probabilistic learning algorithm. We analyzed geo-tagged tweets from New York City over two months to demonstrate the possibilities of social media-powered individual monitors. The capacity to self-monitor and react to impulses and the transmission of information from a wide range of physical devices powers smart city initiatives. The paper advances a theoretical framework to understand AI, specifically in urban contexts. It develops the concept of urban artificial intelligence, capturing the main manifestations of AI in cities. It examines the case of Masdar City, an Emirati urban experiment, to show how the genesis of urban AI is part of a long-standing process of technological development [29]. The study offers new information about how AI might help cities become smarter. As the methodologic strategy, a thorough evaluation of the literature is chosen. The main components of smart city development—economy, society, environment, and governance—are used to categorize the results [30].

### 3.1. Extracted Features

The extremely valuable and significant features are assembled from the literature, as shown in Table 1.

### 3.2. Selected Features

**Figure 1:**
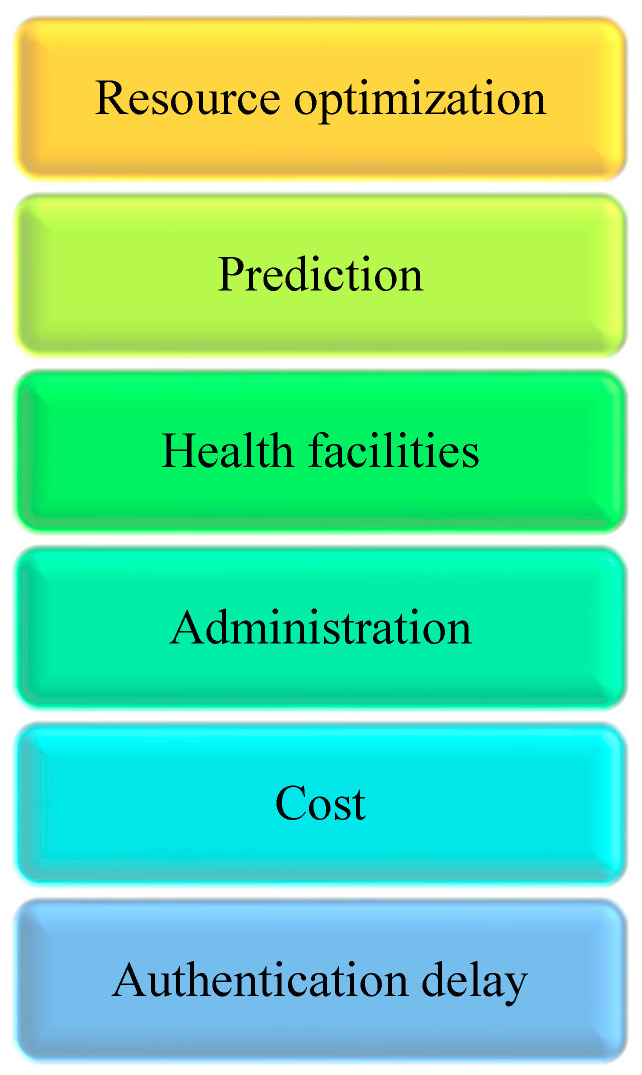
Selected Features.

The following essential traits are revealed by a comprehensive analysis of the literature, as shown in Figure 1. These features were considered as they are the most common features used in the literature and people demonstrate greater preference for them.

### 3.3. AHP Methodology

This computational mathematics approach works well for managing and analyzing challenging decisions. AHP offers a practical method for handling various problems in various circumstances, while coming to wise and practical conclusions. Using the scale Saaty created, which is depicted in Table 2, we may make informed decisions among the numerous options that are accessible, in light of several considerations. In 1980, Saaty originally proposed this tactic [31].

**Figure 2:**
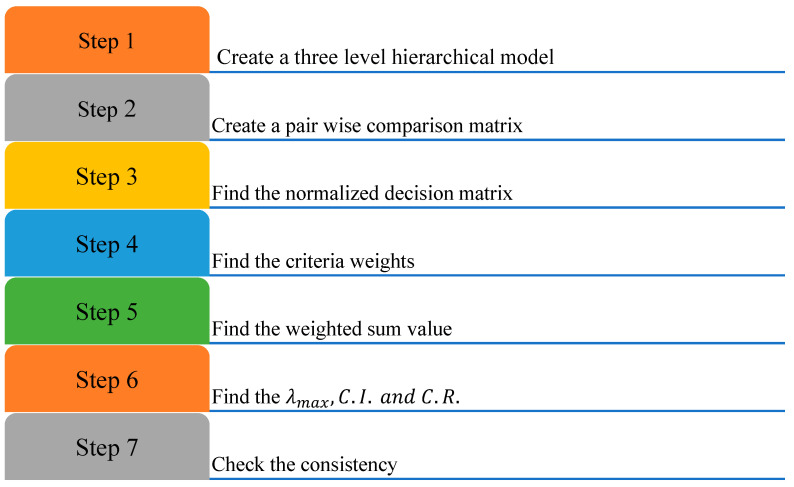
AHP.

The several underpinning principles of the AHP approach are shown in Figure 2.

**Figure 3:**
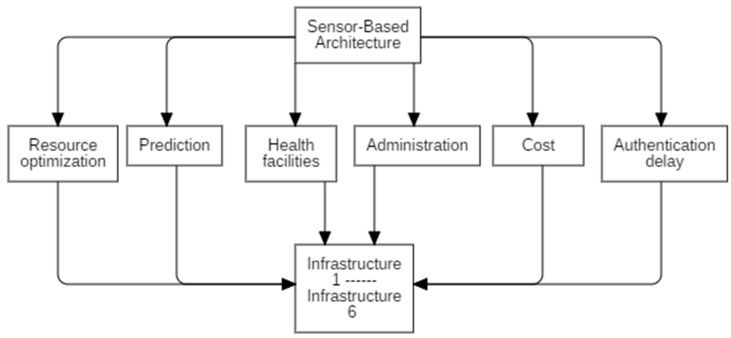
Hierarchical Diagram.

**AHP Tree Diagram.** This diagram presents the issue as a hierarchical structure with three levels. The first level displays the objective, while the second level displays the criteria. The third level, as shown in Figure 3, explains the potential outcomes. The figure shows the goal, criteria, and alternatives for the proposed study. There are total of six features and alternaties.

**Pair-Wise Comparison Matrix.** By providing each attribute with a precise score on the Saaty scale, based on the needs of the person in charge, a matrix is created. The pair-wise comparison matrix for the current condition is shown in Table 3.

**Normalized Matrix.** As shown in Table 4, the required normalized matrix may be calculated using Equation (1). The intial score was given to each criterion and alternative, after which the pairwise comparison was carried out. The various infrastructures included infrastructure 1 to infrastructure 6. These show the different situations for which the pairwise comparisons were carried out.

(1) $$A_{ij}=\frac{X_{ij}}{sum\;of\;each\;column}$$

**Criteria Weights.** As shown in Table 5, the average of each row of the normalized pair-wise comparison matrix is used to determine each criterion’s weight (2).

(2) $$C.W.=\frac{\sum X_{ij\;}}{n}$$

**Consistency Index.** C.I. may be calculated through the use of Equation (3).

(3) $$\begin{array}{c} C.I=\frac{\lambda_{max}-n}{n-1}\\ C.I=\frac{7.29572-6}{6-1}\\ C.I=\;0.25914 \end{array}$$

**Consistency Ratio.** Equation (4) was used to calculate an estimate of the value of C.R.

(4) $$C.R=\frac{C.I}{R.I}$$

These equations show the consistency index and the consistency ratio for the study, for pairwise comparisons.

**Figure 4:**
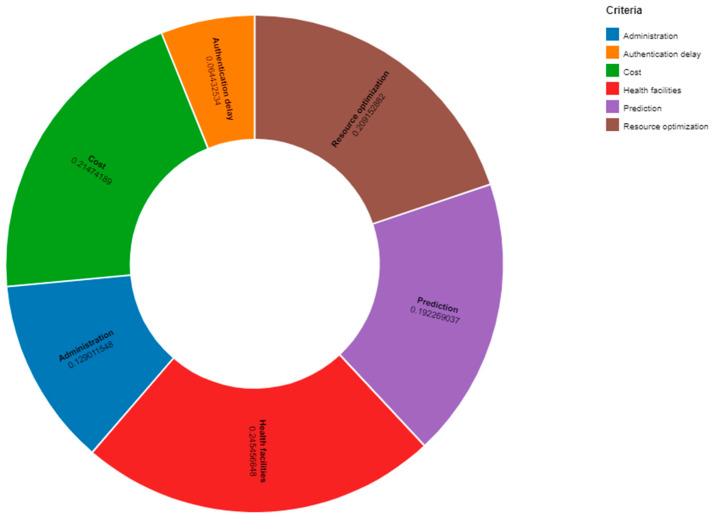
Calculated Weights.

**Resultant Weights.** The results of the attributes assessed using the analytical hierarchy process are shown in Figure 4.

### 3.4. MOORA Method

Each component of the decision matrix is first changed in MOORA to produce the regular lattice, as shown in Table 6. Then, network nodes are constructed for each attribute. The base stations have the highest benefit-type measured values and the lowest cost-type parameter value. The aggregation of factors and biases for the cost type of parameter must be subtracted from the aggregate of weights and biases for the benefit type of criteria to get the evaluation values. Depending on these assessment scores, the choices are ultimately rated. The architectural depiction of the judgment matrix is shown in (5) [32]. This matrix shows the process of pairwise comparisons for each feature, along with its alternative.

(5) $$A=\left[\begin{array}{ccc} a_{11} & a_{12} & \begin{array}{cc} \ldots & a_{1n} \end{array}\\ a_{21} & a_{22} & \begin{array}{cc} \ldots & a_{2n} \end{array}\\ \begin{array}{c} \vdots \\ a_{m1} \end{array} & \begin{array}{c} \vdots \\ a_{m2} \end{array} & \begin{array}{cc} \begin{array}{c} \ldots \\ \ldots \end{array} & \begin{array}{c} \vdots \\ a_{mn} \end{array} \end{array} \end{array}\right]$$

**Figure 5:**
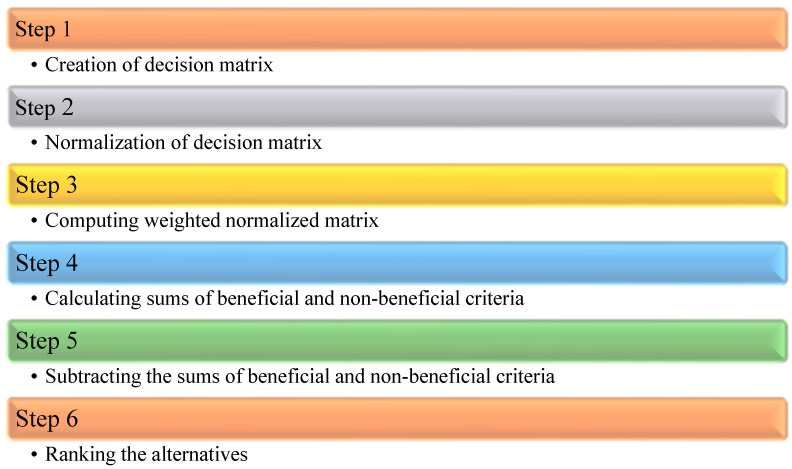
MOORA.

**Flow of MOORA.** The MOORA procedure adhered to the following ideas, as shown in Figure 5.

**Normalized Matrix.** A normalized choice matrix was produced using Equation (6), as shown in Table 7.

(6) $$X_{ij}^{*}=\frac{x_{ij}}{\sqrt{\sum_{i=1}^{m}x_{ij}^{2}}}$$

**Beneficial and Non-beneficial Features.** The total of the favorable and unfavorable characteristics is calculated using Equations (7) and (8), as shown in Table 8.

(7) $$sum\;of\;beneficial\;parameter=y_{i}^{+}=\overset{g}{\underset{j=1}{\sum }}w_{j}x_{ij}^{*}$$

(8) $$sum\;of\;non-beneficial\;parameter=y_{i}^{-}=\overset{n}{\underset{j=g+1}{\sum }}w_{j}x_{ij}^{*}$$

**Ranking.** The choices were determined on the basis of the difference between using Equation (7) or the MOORA technique (8). The candidate with the greatest difference value was ranked first, then the next contender, as shown in Table 9.

## 4. Results and Discussion

**Figure 6:**
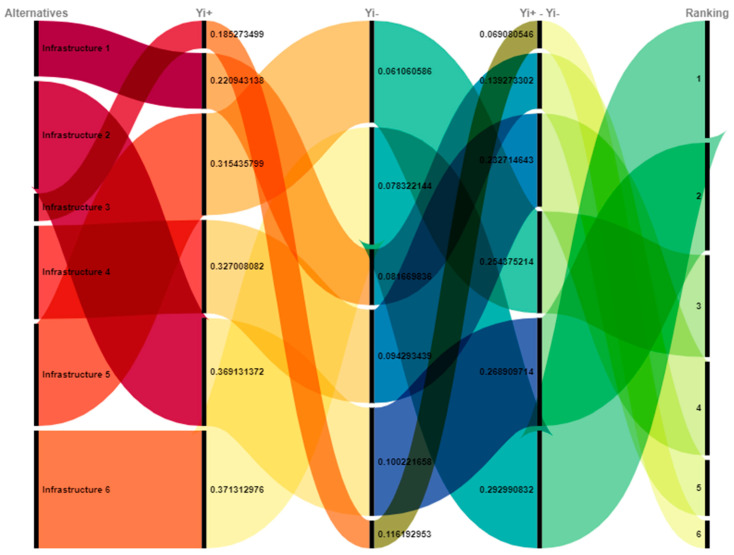
Ranking.

The world is changing very rapidly with the development of modern information and communication technologies. The implementation of automation is the focus of every sector of our lives. To achieve efficiency and feasibility in daily activities, the Internet of Things, along with other technologies, is revolutionizing every task. Smart and intelligent sensors are used for the collection of data related to various parameters such as health, environment, and energy. This article provides an analysis of the contributions made by clever sensing technologies in integration with artificial intelligence and machine learning for effective monitoring and management procedures. The significant use of sensors in smart cities may help provide inhabitants with quality healthcare options. The Internet of Things is discussed from the standpoint of certain applications and services, such as automation and intelligent surveillance. Smart and automated sensors that are based on the Internet of Things are making the concept of intelligent cities a reality. The available literature has many important qualities, the most important of which have been selected to rank the alternatives. The weights of these qualities have been determined using the analytical hierarchy process (AHP), and the rankings were determined using the multi-objective optimization on the basis of research analysis (MOORA) method. Figure 6 shows that, in terms of statistics, Infrastructure 6 was rated at the top and Infrastructure 3 at the bottom.

**Figure 7:**
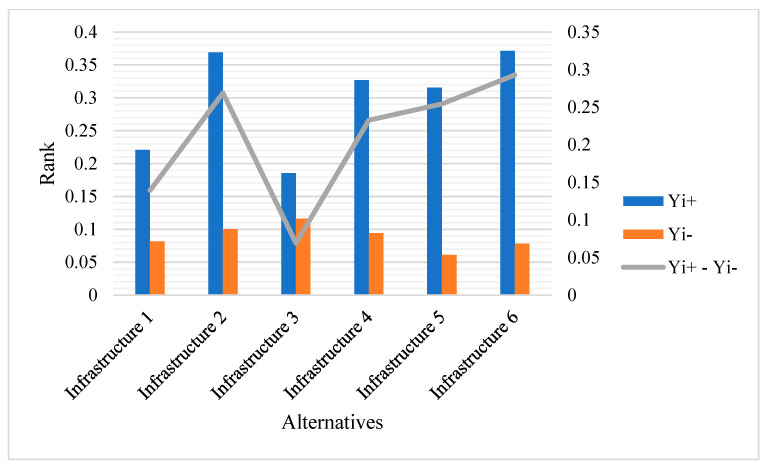
Relationship among alternatives.

Figure 7 displays the overall relationship among the chosen alternatives as determined by the research design.

## 5. Conclusions

By examining a variety of purposes and defining additional features and concepts for sensors and detecting platforms used in green infrastructure, power networks, and electric grids, this article’s ultimate objective was to provide readers with a better understanding of the existing techniques in this field. The sensors-grounded paradigms are very beneficial for use in vehicles for avoiding any unwanted situation and can also be used for navigation purposes. They have numerous applications in the health sector, including smart monitoring, treatment, and pandemic prevention and control. For the positive consumption of energy resources and other assets, sensors can be employed with smart checking and analytics. A problem that commonly occurs in our cities is the inability to find open parking places. People who commute to work are looking for a space to park, clogging the roads. The most common urban problems, such as parking availability and traffic congestion, are being solved through IoT. Allocating scores to the chosen qualities is accomplished using the multi-criteria decision making (MCDM) approach known as AHP, while the ranking is accomplished using the MOORA methodology. The current study took into account a thorough listing of the publications that are available in the field of investigation. The investigation found different approaches in the literature survey, which suggests that academics might determine fresh approaches in the sector.

## Figures and Tables

**Table 1:** Extracted Features.

Citations	Features	Citations	Features
[1]	Automation, systems’ performance, energy utilization	[10]	Parking allocation, reliability, productivity
[2]	resource optimization, communication, efficiency, monitoring	[11]	e-voting, authentication delay, blockchain, transparency
[3]	Complex sensing, flexibility, scalability, cost reduction	[12]	Monitoring, safety, power usage, automation
[4]	Management, monitoring, controlling, safety	[13]	Vehicle automation, controlling, real-time, privacy
[5]	Clean atmosphere, location finding, real-time, effective administration	[14]	Power grid, services, performance
[6]	Data analytics, big data, predictions	[15]	Smart lighting, energy consumption, cost
[7]	Performance, sustainaibility, management	[16]	Smart parking, reservation, navigation
[8]	Accuracy, stability, hardware testing	[19]	Motion sensor, users’ services, smart city
[9]	Security, cloud computing, big data, services	[20]	Health facilities, navigation, interactivity

**Table 2:** Saaty scale.

Scale	Definition
1	Equal Importance
3	Moderate Importance
5	Essential or Strong Importance
7	Very Strong Importance
9	Extreme Importance
2, 4, 6, 8	Intermediate Values

**Table 3:** Pair-Wise Comparison Matrix.

	Resource Optimization	Prediction	Health Facilities	Administration	Cost	Authentication Delay
Resource optimization	1	3	0.33	2	2	2
Prediction	0.33	1	3	2	0.33	2
Health facilities	3	0.33	1	2	2	3
Administration	0.5	0.5	0.5	1	2	2
cost	0.5	3	0.5	0.5	1	3
Authentication delay	0.5	0.5	0.33	0.5	0.33	1
sum	5.83	8.33	5.66	8	7.66	13

**Table 4:** Normalized Matrix.

	Resource Optimization	Prediction	Health Facilities	Administration	Cost	Authentication Delay
Infrastructure 1	0.171527	0.360144	0.058304	0.25	0.261097	0.153846
Infrastructure 2	0.056604	0.120048	0.530035	0.25	0.043081	0.153846
Infrastructure 3	0.51458	0.039616	0.176678	0.25	0.261097	0.230769
Infrastructure 4	0.085763	0.060024	0.088339	0.125	0.261097	0.153846
Infrastructure 5	0.085763	0.360144	0.088339	0.0625	0.130548	0.230769
Infrastructure 6	0.085763	0.060024	0.058304	0.0625	0.043081	0.076923
sum	1	1	1	1	1	1

**Table 5:** Criteria Weights.

	Resource Optimization	Prediction	Health Facilities	Administration	Cost	Authentication Delay	C.W.
Infrastructure 1	0.171527	0.360144	0.058304	0.25	0.261097	0.153846	0.209153
Infrastructure 2	0.056604	0.120048	0.530035	0.25	0.043081	0.153846	0.192269
Infrastructure 3	0.51458	0.039616	0.176678	0.25	0.261097	0.230769	0.245457
Infrastructure 4	0.085763	0.060024	0.088339	0.125	0.261097	0.153846	0.129012
Infrastructure 5	0.085763	0.360144	0.088339	0.0625	0.130548	0.230769	0.159677
Infrastructure 6	0.085763	0.060024	0.058304	0.0625	0.043081	0.076923	0.064433

**Table 6:** Decision Matrix.

	Resource Optimization	Prediction	Health Facilities	Administration	Cost	Authentication Delay
Infrastructure 1	2	3	4	7	5	4
Infrastructure 2	7	8	7	4	5	8
Infrastructure 3	3	2	5	3	7	6
Infrastructure 4	6	6	7	4	6	4
Infrastructure 5	7	6	7	2	3	5
Infrastructure 6	9	6	8	3	4	6

**Table 7:** Normalized Matrix.

	Resource Optimization	Prediction	Health Facilities	Administration	Cost	Authentication Delay
Infrastructure 1	0.132453	0.22056439	0.25197632	0.68973049	0.39528471	0.2879263
Infrastructure 2	0.463586	0.5881717	0.44095855	0.39413171	0.39528471	0.5758526
Infrastructure 3	0.19868	0.14704292	0.31497039	0.29559878	0.55339859	0.43188945
Infrastructure 4	0.39736	0.44112877	0.44095855	0.39413171	0.47434165	0.2879263
Infrastructure 5	0.463586	0.44112877	0.44095855	0.19706586	0.23717082	0.35990788
Infrastructure 6	0.59604	0.44112877	0.50395263	0.29559878	0.31622777	0.43188945

**Table 8:** Beneficial and Non-beneficial Features.

Alternatives	Yi+	Yi−
Infrastructure 1	0.220943	0.08167
Infrastructure 2	0.369131	0.100222
Infrastructure 3	0.185273	0.116193
Infrastructure 4	0.327008	0.094293
Infrastructure 5	0.315436	0.061061
Infrastructure 6	0.371313	0.078322

**Table 9:** Ranking of the Alternatives.

Alternatives	Yi+	Yi−	Yi+-Yi−	Ranking
Infrastructure 1	0.220943	0.08167	0.139273	5
Infrastructure 2	0.369131	0.100222	0.26891	2
Infrastructure 3	0.185273	0.116193	0.069081	6
Infrastructure 4	0.327008	0.094293	0.232715	4
Infrastructure 5	0.315436	0.061061	0.254375	3
Infrastructure 6	0.371313	0.078322	0.292991	1

## Data Availability

Not applicable.
